# The Emerging Role of GLP-1 Receptors in DNA Repair: Implications in Neurological Disorders

**DOI:** 10.3390/ijms18091861

**Published:** 2017-08-26

**Authors:** Jenq-Lin Yang, Wei-Yu Chen, Shang-Der Chen

**Affiliations:** 1 Institute for Translation Research in Biomedicine, Kaohsiung Chang Gung Memorial Hospital, 123 Dapi Road, Kaohsiung 83301, Taiwan; jyang@adm.cgmh.org.tw (J.-L.Y.); wychen624@cgmh.org.tw (W.-Y.C.); 2 Department of Neurology, Kaohsiung Chang Gung Memorial Hospital, 123 Dapi Road, Kaohsiung 83301, Taiwan; 3 College of Medicine, Chang Gung University, 259 Wenhua 1st Road, Taoyuan 33302, Taiwan

**Keywords:** neurodegenerative disorders, DNA damage and repair, apurinic/apyrimidinic endonuclease 1 (APE1), glucagon-like peptide 1 receptor (GLP-1R)

## Abstract

Glucagon-like peptide-1 (GLP-1) is originally found as a metabolic hormone (incretin) that is able to regulate blood-glucose levels via promoting synthesis and secretion of insulin. GLP-1 and many analogues are approved for treatment of type II diabetes. Accumulating results imply that GLP-1 performs multiple functions in various tissues and organs beyond regulation of blood-glucose. The neuroprotective function of GLP-1 has been extensively explored during the past two decades. Three of our previous studies have shown that apurinic/apyrimidinic endonuclease 1 (APE1) is the only protein of the base excision repair (BER) pathway able to be regulated by oxidative stress or exogenous stimulations in rat primary cortical neurons. In this article, we review the role of APE1 in neurodegenerative diseases and its relationship to neuroprotective mechanisms of the activated GLP-1 receptor (GLP-1R) in neurodegenerative disorders. The purpose of this article is to provide new insight, from the aspect of DNA damage and repair, for studying potential treatments in neurodegenerative diseases.

## 1. Introduction

The genetic material of organisms, including prokaryotes and eukaryotes, are constantly attacked by numerous endogenous metabolites and exogenous agents. Both the endogenous and exogenous insults lead to various types of DNA damage, including abasic sites, oxidized base modification, deamination, methylation, nucleotide deletion, nucleotide insertion, bulky abducts, single-strand breaks (SSBs), double-strand breaks (DSBs), inter- and intra-strand cross-links (ICLs), and DNA-protein cross-links [1,2]. It is well known that unrepaired nuclear DNA damage is able to induce mutations and chromosomal aberrations that lead to disruption of transcription and replication, causing cellular dysfunction or oncogenic transformation, cellular senescence, aging, and initiation of programmed cell death [3]. The majority of mutations in human tissues are of endogenous origin but are also compromised by various environmental factors or exogenous toxic agents. For instance, biological molecules are vulnerable to spontaneous chemical reactions, mostly from hydrolysis and the enzymatic reactions may have an inevitable error rate which may result in overproduction of reactive oxygen and nitrogen species and cause harmful effects on other biological molecules. Moreover, environment factors—Including numerous chemicals, ultraviolet (UV) radiation, and X-rays—continuously damage cellular structures including DNA [4]. As time proceeds, those factors may contribute to the development of cancer and other diseases [4]. 

In response to DNA damage, organisms have developed copious and overlapping repair mechanisms to eliminate threats to DNA integrity. In mammalian cells, several predominant DNA repair mechanisms are well studied, including direct repair, base excision repair (BER), nucleotide excision repair (NER), mismatch repair, homologous recombination repair (HR), and non-homologous end-joining repair (NHEJ) [5,6]. Failures or defects in DNA repair mechanisms have been associated with many human diseases, such as cancer, progeria, and neurodegenerative disorders [7,8,9,10,11].

Several strategies are proposed to restore neurological function or to ameliorate neuronal injury, especially for neurodegenerative diseases. The pathogenesis of neurodegenerative disorders are commonly seen as oxidative stress, protein aggregation, inflammation, neuronal death, and DNA damage. Potential therapeutic approaches via counteracting oxidative stress [12,13], inflammatory responses [14,15], protein misfolding [16,17], and apoptosis [17,18] were extensively studied, but repair of damaged DNA is rarely mentioned. Nevertheless, accumulation of nuclear and mitochondrial DNA damage has been observed in brain regions correlated with neurodegenerative diseases [19,20]; therefore, DNA repair mechanisms have been suggested to play a pivotal role in maintaining genomic integrity and cell survival, as well as being potential candidates for innovative approaches for prevention and treatment of neurodegenerative diseases. Oxidative stress is viewed as one of the major threats for developing neuronal DNA damage and the efficiency of DNA repair is a critical and distinctive factor affecting neurodegenerative diseases [21]. BER is a primary repair mechanism for oxidized DNA bases and single-strand breaks in nuclear and mitochondrial DNA in eukaryotic cells [22,23]. In addition, defects in BER are linked to some human disease syndromes and age-related neurodegenerative diseases [24]. Our previous studies demonstrated that apurinic/apyrimidinic endonuclease 1 (APE1) is the only protein of the BER pathway upregulated after glutamate-induced oxidative stress [25], as well as treatments with brain-derived neurotrophic factor (BDNF) and glucagon-like peptide-1 (GLP-1) in rat primary cortical neurons [26,27]. It was also demonstrated that knockdown APE1 can induce accumulation of oxidative DNA damage with glutamate treatment and selective depletion of cyclic AMP (cAMP)-response element-binding protein (CREB) by siRNA can prevent glutamate-induced upregulation of APE1 [25]. These studies reported that the DNA repair efficiency of BER is enhanced by upregulated APE1. Enhancing APE1 expression could also be beneficial for reactive oxygen species (ROS)-related neurodegenerative diseases. 

One of the emerging candidates for treating neuronal injury, both acute and chronic, is the GLP-1 receptor (GLP-1R). In addition to the treatment of adult type II diabetes, stimulation of the GLP-1R has also been reported to exert neuroprotective effects in various experimental models of cerebral ischemia, traumatic brain injury, Alzheimer’s disease (AD), Parkinson’s disease (PD), Huntington’s disease (HD), amyotrophic lateral sclerosis (ALS), multiple sclerosis (MS), and many other neurodegenerative diseases [28,29,30,31,32,33,34]. GLP-1R are also expressed throughout the brain and GLP-1 agonists can exert characteristics which are independent of pancreatic effects on glucose control and can cross over the blood–brain barrier (BBB) to influence several cellular pathways with respect to counteracting inflammation, oxidative stress, and apoptosis within the central nervous system. In our recent study, we reported that stimulation of GLP-1R enhanced APE1 expression and BER activity; moreover, administration of Exendin-4 (EX-4), a GLP-1R agonist, substantially reduced ischemia-induced nuclear DNA damage in brain cells [26]. 

In this review, we discuss the protective effects of GLP-1R that correlate with the BER pathway in cerebral ischemic strokes, brain trauma, and chronic neurodegenerative diseases, and emphasize potential therapeutic strategies utilizing DNA repair mechanisms. 

## 2. DNA Damage and Repair in Neurological Disorders

DNA is an unstable molecule compared to other intracellular molecules in organisms. Thus, maintaining genomic function and integrity is an emerging issue in studying the pathology of diseases. In mitotic cells, the cell cycle machinery plays a major role in response to DNA damage. It can eliminate the damaged DNA through a complex set of DNA repair mechanisms and preserve genomic integrity, or eradicate seriously injured cells through apoptotic processes [35,36,37]. Consequently, the mechanisms of response and repair to DNA damage are also closely integrated with regulation of the cell cycle, DNA replication, and transcription. Several proteins are shared between the DNA damage response and repair pathways and other cellular processes utilizing DNA polymerases, RNA polymerases, and transcription factors [38]. On the contrary, terminally differentiated cells are characterized by permanent withdrawal from the cell cycle, without genomic replication; the mitotic cell-associated DNA repair pathways, such as HR and global genomic nucleotide excision repair (GGNER, also named global genomic repair (GGR)), are inactive or deficient [39,40,41].

It is known that the average brain mass accounts for about 2% body weight of an adult human, but the relatively small size organ consumes 20% of oxygen due to high rate of metabolism from varying mental and motoric activities [42]. The highly oxygenized and metabolic environment of the brain likely leads to excessive oxidative stress and causes damage to various components of the brain. Over the past two decades, extensive study and increasing evidence has revealed that brain tissues are susceptible to oxidative DNA damage which may lead to the pathogenesis of neurodegenerative diseases and is tightly connected to the competence of DNA repair [1,43,44].

Neurons are unique cells in the brain that are highly active and terminally differentiated with long, out-reaching neurites for constantly transmitting neuroelectric signals. It is well known that neurons, with a high rate of oxidative metabolism and relatively low levels of antioxidant enzymes, are highly susceptible to oxidative stress [45]. The excessive ROSs attack various cellular components, including proteins, lipids, and DNA, eventually causing cell death [46,47]. Post-mitotic neurons do not replicate their genomic DNA and tend to produce ROS; nevertheless, removing oxidatively-damaged nucleotides and maintaining genomic integrity become crucial issues for neuronal function and survival. BER is the primary DNA repair mechanism to remove oxidized bases, deamination, methylation, alkylation, and SSBs [1,2]. The BER pathway involves multiple enzymatic steps, starting with the identification and elimination of a damaged base by a DNA glycosylase, incising the sugar phosphate backbone by a DNA endonuclease, filling in the corresponding base by a DNA polymerase, and ligating the DNA nick by a DNA ligase [48]. APE1 is the most abundant protein among BER enzymes in human cells, which incises the sugar phosphate backbone forming a nick break and plays an essential role in the BER pathway in response to oxidative DNA damage [22]. Besides its endonuclease function, APE1 also has a redox effector role in promoting the DNA-binding activity of activator protein 1 (AP-1), p53, nuclear factor-κB (NF-κB), hypoxia-inducible factor 1 α (HIF-1α), and paired box 5 (PAX5) [49,50,51]. Due to multifunctional roles, APE1 has been implicated to play a role in various human diseases, including neurodegeneration or other neurological disorders linked with alterations in the expression, subcellular localization, and other activities. Thus, APE1 is a potential target for therapeutic intervention by various agents that can modulate its expression and functions [52].

Downregulation of APE1 expression or suppression of its endonuclease activity could modulate vulnerability toward assorted types of cancer or cardiovascular diseases [53,54,55], as well as neurological disorders, including AD [55,56,57,58,59], PD [60], HD [61], ALS [62], traumatic brain injury [63], and cerebral ischemia [64]. All evidence in previous studies underscored the pivotal role of APE1 in various human diseases and suggested that this is a promising therapeutic research area for their treatment and management. We review APE1 expression, which correlates with various neurological diseases, in the next section.

### 2.1. Apurinic/Apyrimidinic Endonuclease 1 (APE1) and Cerebral Ischemic Stroke

Energy and oxygen depletion in neuronal cells is a hallmark of strokes and other ischemic or hypoxic brain injuries. A study of primary neurons by Singh and Englander reported that diminution of cellular ATP was accompanied by depletion of nuclear APE1 and was associated with neuronal death, but other BER proteins were not affected. Singh and Englander obtained similar results from in vivo studies. The results suggested that disrupted energy homeostasis triggered nuclear APE1 relocation, and consequently, induced neuronal death [65]. Another study by Fujimura et al. [66] showed that APE1 rapidly decreases, leading to overwhelming DNA fragmentation in infarcted regions displaying necrotic or apoptotic brain cell death after transient focal ischemia. Fujimura’s results implied that the rapid decline of APE1 expression impairs the DNA repair mechanism and contributes to neuronal necrosis or apoptosis after photothrombotic cerebral ischemia [66]. Deficiency of APE1 enzymatic activity is also suggested to be a risk factor or a detrimental factor in ischemic stroke. The study by Naganuma et al. on single-nucleotide polymorphisms (SNPs) of APE1 suggested that APE1 is a susceptibility gene for cerebral infarction and the G-C-T haplotype (haplotype is a term of SNP and means a group of genes was inherited together from a single parent) of APE1 may be a genetic marker for cerebral infarct in humans [67]. Leak et al. reported that enhanced APE1 expression, either through an increase of the endogenous protein or through transgene overexpression, protected neuronal structures, DNA integrity, synaptic function, and behavioral status from transient global ischemia in rats [68]. A recent study by Stetler et al. [69] showed that induced deletion of APE1 significantly increased the infarct area, impaired the recovery of motor activity and cognitive function, and augmented neuron and oligodendrocyte degeneration in APE1 conditional knockout (cKO) stroke mice. The results also demonstrated that endogenous APE1 protects against ischemic infarction in both gray and white matter and facilitates the functional recovery of the central nervous system after stroke injury [69]. Furthermore, APE1 cKO increased abasic sites and activated pro-death signaling proteins, such as the p53 upregulated modulator of apoptosis (PUMA) and poly (ADP-ribose) polymerase-1 (PARP1), after transient focal ischemia [69]. In our recent study, we showed that enhancing DNA repair activity by elevating the expression of APE1 exerts protective effects in the neuronal cells of ischemic stroke rats [26].

### 2.2. APE1 and Traumatic Brain Injury

The common causes of traumatic brain injury (TBI) include traffic accidents, sporting activities, and violent incidences. Primary brain injury relates to the damage that occurs at the time of the trauma, when tissues and blood vessels are impacted, compressed, and distorted. Secondary, or post-traumatic, injury is caused by a complex set of cellular and biochemical cascades that worsen the primary injury, accounting for the greatest number of TBI deaths occurring in hospitals [70]. Molecular effects of TBI include dysregulation of the blood–brain barrier, release of factors that cause inflammation, free radical overload, excessive release of the neurotransmitter glutamate, an influx of calcium and sodium ions, and dysfunction of mitochondria [71]. All of the above pathological mechanisms are potential targets for intervention treatments to prevent further deterioration during TBI. 

As stated above, APE1 is an important BER protein for repairing oxidative DNA damage in both nuclei and mitochondria. Many previous studies reported that secondary injury from TBI is specifically related to an inflammatory response and overwhelming oxidative stress [72,73,74]. Until now, only limited studies have dealt with DNA damage and repair and APE1 expression or activity in traumatic brain injury. The study of Morita-Fujimura et al. was the first to report that APE1 rapidly decreases after cold injury-induced brain trauma and their results also suggest that the early decrease of APE1 is correlated with DNA repair failure, thereby contributing to DNA-damage-induced neuronal cell death [63]. Lewén et al. reported that reduction of APE1 was closely correlated with oxidative stress after traumatic brain injury and the decrease of APE1 expression preceded DNA fragmentation [75]. According to Lewén’s finding, APE1 is a reliable marker for oxidative cellular injury.

### 2.3. APE1 and Alzheimer’s Disease

Oxidative stress is one of the major pathological components of neurodegenerative diseases, causing systemic cellular damage involving oxidized lipids, proteins, RNA, and DNA [76,77]. The oxidative DNA repair pathway, BER, is tightly bound to the survival fate of neurons. A recent study by Lillenes et al. showed that mRNA levels of APE1 were significantly lower in the entorhinal cortex of AD patients than in the same cortical regions of healthy controls [78]. The results of Lillenes’ study revealed that alterations in BER gene expression is an antecedent to AD occurrence and connects DNA repair in the brain to the etiology of AD at the molecular level. The nuclear APE1 level was found to increase in the AD cerebral cortex by immunostaining [59]. This finding supports the view that DNA damage is involved in the pathogenesis of AD. The findings of Maynard et al. showed that mononuclear cells of peripheral blood had lower APE1 activity and reduced DNA repair efficiency in AD patients and that these biochemical activities are reliable biomarkers for AD [79].

The multifunctional APE1 molecule also has neuronal protective roles in connection with AD beyond DNA repair. An in vitro study by Mantha et al. demonstrated that APE1 is associated with cytoskeleton elements, energy-related metabolic enzymes, stress-responsive proteins, and heterogeneous nuclear ribonucleoprotein H, providing protective effects in Aβ_25–35_-treated PC12 and SH-SY5Y cell lines [58]. Another study by Mantha et al. [80] also showed that Aβ_25–35_-treated neuroblastoma cells produced higher levels of ROS and reactive nitrogen species (RNS), and lower levels of mitochondrial APE1, as well as lower activities in respiratory complexes (I, III, and IV). However, enhancing APE1 expression effectively increased oxidative phosphorylation and decreased ROS/RNS production [80]. All lines of evidence suggest that APE1 plays a neuroprotective role and is a therapeutic target for AD.

### 2.4. APE1 and Parkinson’s Disease

PD is the second most prevalent neurodegenerative disease and shares similar pathogenesis with other neurodegenerative disorders in dopaminergic neuron, including oxidative stress, chronic inflammatory responses, protein aggregation, oxidative DNA damage, and dopaminergic neuronal death. With these mechanisms, dysfunctional mitochondria and oxidative stress are major components of the pathogenesis of PD. Maintaining genomic integrity against oxidative insults is among the most important issue for neuronal survival. Hence, BER proteins have been suggested to be good candidates for therapeutic intervention. Gencer et al. investigated the gene polymorphisms of BER proteins in PD patients and healthy controls, and suggested that *APE1,* X-ray repair cross-complementing protein 1 *(XRCC1),* and X-ray repair cross-complementing protein 1 *(XRCC3)* genetic variants may be risk factors for developing PD, due to loss of dopaminergic neurons in the substantia nigra and locus coeruleus [60]. Overexpression of APE1 significantly suppressed ROS levels, increased cell viability, and inhibited apoptosis in PC12 cells treated with MPP+ (1-methyl-4-phenylpyridinium). Knockdown of APE1 showed the opposite effects [81]. The recent study of Scott et al. reported that modulation of Parkin and PTEN-induced putative kinase 1 (PINK1) activities under oxidative stress caused a decrease of endogenous APE1 in SH-SY5Y, HEK293, and A549 cells [82]. Parkin, a PD associated gene, is an E3 ubiquitin ligase and plays a crucial role in mitophagy. The role of Parkin in regulating DNA repair proteins has not been clearly elucidated. However, it is well known that dysfunctional mitochondria, oxidative stress, and oxidative DNA damage are tightly linked together. The results of the above studies support the notion that a deficiency in the APE1 protein or activity is involved in the pathogenesis of PD, while enhancement of APE1 expression or activity remarkably increases neuronal viability. 

### 2.5. APE1 and Huntington’s Disease

Huntington’s disease (HD) is an inherited, autosomal dominant neurodegenerative disease caused by trinucleotide (CAG) repeat expansion in the 5′-end of the *huntingtin* (*htt*) gene. In addition, HD is characterized by a progressive defect of motor and cognitive functions. Aggregated mutant huntingtin protein elevates *N*-methyl-d-asparate (NMDA) activity and disrupts Ca^2+^ homeostasis, inducing mitochondrial dysfunction which results in the generation of ROS [83,84]. Higher levels of oxidative nuclear DNA and mitochondrial DNA damage were found in brain samples of both the HD mouse model and HD patients [85,86]. It is well known that striatal cells with mutant *htt* show higher basal levels of mitochondrial-generated ROS, lower spare respiratory capacity, and more mitochondrial DNA (mtDNA) lesions. A similar phenomenon—higher ROS levels, significant mtDNA damage and mtDNA depletion, and a significant decrease in spare respiratory capacity—have been observed in human HD striata and HD skin fibroblasts. Furthermore, the level of mitochondrial APE1 is higher in wild-type (Q7) striatal cells than mutant *htt* cells after an oxidative insult [61]. All of these results indicate that APE1 is an important target in the maintenance of mitochondrial DNA integrity and function in HD treatment.

## 3. The Glucagon-Like Peptide-1 Receptor (GLP-1R) in Neurological Disorders

**Figure 1:**
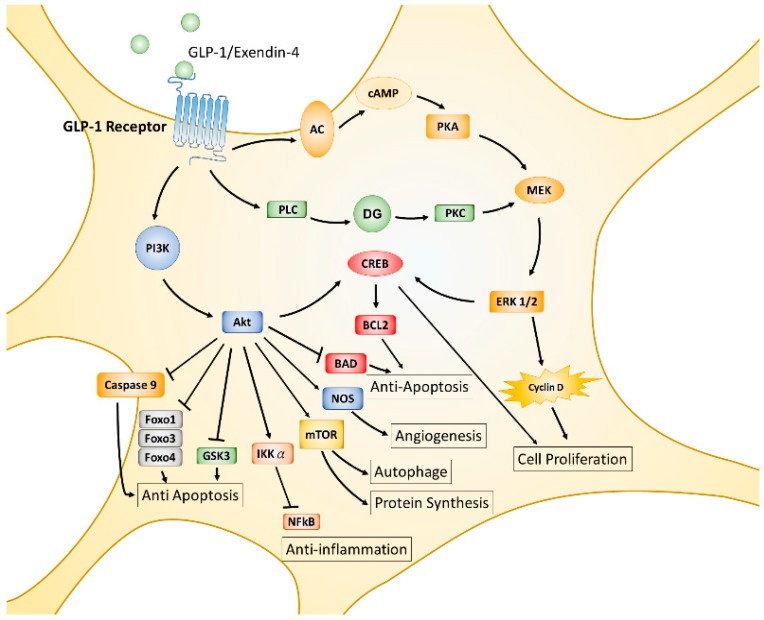
The diagram illustrates downstream signaling pathways and cellular functions after activation of the GLP-1 receptor. Stimulation of GLP-1R triggers two major signaling pathways: PI3K-AKT and MEK-ERK1/2. Consequently, two signaling axes work on phosphorylating or activating CREB. Both signaling pathways lead to the cellular processes of anti-apoptosis, anti-inflammation, growth, autophagy, angiogenesis, and proliferation. Most downstream responses of activated GLP-1R provide cellular protective functions. ┤: downregulation.

The incretin hormone GLP-1, is an endogenous 30-amino acid multifunctional peptide hormone secreted from enteroendocrine L-cells of the small intestine in response to food intake, reported in 1985 by the Creutzfeldt group [87]. GLP-1 promotes glucose-induced insulin biosynthesis and secretion, as well as inhibiting glucagon secretion for maintaining glucose homeostasis. GLP-1 also exerts trophic effects, such as triggering islet β cell proliferation, differentiation, inhibiting apoptosis, and enhancing cell survival [88,89]. The actions of GLP-1 or its analogues are mediated by the GLP-1 receptor (GLP-1R), a seven-transmembrane spanning G protein-coupled receptor. The activated α/β/γ subunit of the G protein complex directly activates phosphoinositide 3-kinase (PI3K), adenyl cyclase (AC), and phospholipase C (PLC), then leads to further phosphorylation and activation of various downstream signaling pathways, including AC-PKA (protein kinase A)-MEK (mitogen-associated protein kinase kinase)-ERK (extracellular signal-regulated kinase), PLC- protein kinase C (PKC)-MEK-ERK, and PI3K- protein kinase B (PKB or also called AKT) [90,91,92] (displayed in Figure 1). GLP-1R is initially found expressing in pancreatic islets cells and activated GLP-1R induces insulin synthesis and release; thus, GLP-1R agonists or dipeptidyl peptidase 4 (DPP-4) inhibitors have been used in the treatment of type 2 diabetes mellitus (T2DM) [93]. It was noted that GLP-1R is also expressed throughout the brain, including in the frontal cortex, hypothalamus, thalamus, hippocampus, cerebellum, and substantia nigra [26,94,95]. Accumulating evidence suggests that GLP-1 agonists exert characteristics which are independent of pancreatic effects on glucose control and can cross over the blood–brain barrier (BBB) to influence several cellular pathways with respect to neuroinflammation, mitochondrial function, neuronal protection, and cellular proliferation within the central nervous system (CNS) [96].

The functions of GLP-1 and GLP-1R have been extensively studied in the past two decades. Convincing results from numerous studies indicate that GLP-1 plays multiple protective roles and affects almost all tissues and organs in animal models or humans [97,98,99,100,101,102]. The MEK-ERK and PI3K-AKT pathways are two major downstream signaling axes of activated GLP-1R that predominantly regulate downstream cellular events. AKT is an important hub of downstream signaling for growth factors, hormones, cytokines, and many other cellular stimuli. Each AKT- or MEK-activated substrate or downstream signaling axis results in single or multiple cellular processes such as protein synthesis, cell proliferation, angiogenesis, apoptosis, inflammation, mitochondrial biogenesis, and autophagy. AKT phosphorylates mammalian targets of rapamycin (mTOR), the cAMP response element-binding protein (CREB), and nitrogen oxide synthase (NOS), triggering additional cellular processes, including protein synthesis or growth, autophagy, proliferation, and survival; on the contrary, AKT directly phosphorylates glycogen synthase kinase 3β (GSK-3β), Forkhead transcription factors (Forkhead box protein O1 (FOXO1), FOXO3a, FOXO4), Bcl-2-associated death promoter protein (BAD), caspase 9, and inhibitor kappa B kinase α (IKKα), thereby suppressing downstream cellular functions, including inflammatory responses and apoptotic processes [103,104,105,106,107]. Like the AKT signaling axes, MEK-ERK activated signaling axes also modulate similar cellular processes in response to stimulation of G protein-coupled receptors or tyrosine kinase receptors [108,109,110] (see Figure 1).

Emerging evidence reveals that downstream signaling pathways of GLP-1R play a pivotal role in neuroprotection in various neurological disorders [96,109,111]. The following sections discuss the potential of GLP-1/GLP-1R induced mechanisms in the protection and/or prevention of both acute brain disorders like cerebral ischemia or brain injury, and chronic neurodegenerative diseases such as AD, PD, HD, and ALS.

### 3.1. Stimulation of GLP-1R and Cerebral Ischemic Stroke

Ischemic stroke, an acute hypoxic injury, is the most prevalent type of stroke caused by a brain artery being blocked by a blood clot. The brain cells of the infarct area suffer depletion of oxygen and glucose which leads to cell death and irreversible brain injury, approximately three hours after stroke occurrence. Subsequently, the overwhelming metabolite (lactate) and neurotransmitter (glutamate) are released from cells in the infarct area, interrupting the ion and energy homeostasis and the blood–brain barrier function, as well as inducing excitotoxicity which causes edema and further cell death in the penumbra area, approximately 48–72 h after occurrence of the stroke. Therefore, maintaining cellular homeostasis, cellular function, and cell viability is the most important issue during the critical post-stroke period. Exploring the neuroprotective function of GLP-1 and agonists of the GLP-1R has been a fast growing area of research in the last decade. Long-lasting or DPP-4 resistant GLP-1 analogues and DPP-4 inhibitors are widely studied to test their effect on cerebral ischemic conditions but fewer studies explore the signaling mechanisms that are involved in their neuroprotective functions.

Exendin-4 (EX-4), a long-lasting GLP-1R agonist, reduced infarct volume and cell death by suppressing oxidative stress and the inflammatory response, thus improving functional deficits via the cAMP-mediated pathway, in transient focal cerebral ischemic mice [112]. Two studies in the cerebral ischemic rat model reported that EX-4 exerted neuroprotective functions through AKT-eNOS and cAMP-PKA-CREB, respectively [113,114]. An in vitro study also showed that administration of EX-4 rescued rat cortical neurons from oxygen/glucose deprivation (OGD) through the PKA pathway [108]. Hypoxia-inducible factor-1α (HIF-1α), a transcription factor, plays a key role in the cellular response to hypoxia in mammals. The study of Jin et al. reported that the neuroprotective effect of EX-4 was via regulating HIF-1α expression in a transient global ischemia gerbil model, in OGD human neuroblastoma cells (SH-SY5Y), and in OGD mouse cortical primary neurons [115]. 

Liraglutide, also a long-acting GLP-1 analogue, was reported to have a role in inhibiting cell apoptosis and reducing excessive ROS. It also improved mitochondrial function by activating AKT and ERK pathways and inhibiting phosphorylation of c-Jun-NH2-terminal kinase (JNK) and p38 in neurons in both OGD in vitro and middle cerebral artery occlusion (MCAO) in vivo studies [116]. Another study showed that delayed administration of liraglutide, starting one day after MCAO, still improves metabolic and functional recovery of neurons, astrocytes, and endothelia after cerebral ischemia in rats [117].

GLP-1 can be degraded by DPP-4 in a very short time in the mammalian circulation system. Thus, DPP-4 inhibitors are able to delay the degradation of GLP-1, increase the effective concentration, and reach target organs or tissues. DPP-4 inhibitors have similar neuroprotective functions as GLP-1 and its analogues. Linagliptin and Alogliptin, two DPP-4 inhibitors, significantly reduce infarct volume, increase tolerance to focal cerebral ischemia, and alleviate neurological deficits [118,119]. In addition, Alogliptin is capable of enhancing BDNF production to attenuate cerebral injuries [119]. However, studies have also revealed that Linagliptin enhances neural stem cell proliferation after strokes and the neuroprotective effect may go beyond the well-known GLP-1 receptor function [120].

### 3.2. Stimulation of GLP-1R and Traumatic Brain Injury

Mild traumatic brain injury (mTBI), commonly known as concussion, is the most prevalent traumatic brain injury (TBI) and represents a major public health concern. It is the most frequent incident of mortality and disability in the young population as well as the major cause of morbidity in the elderly [121,122]. Headaches, fatigue, depression, anxiety, irritability, as well as impaired cognitive function are common symptoms of mTBI [123]. No pharmaceutical therapies are currently available to manage the pathological development associated with mTBI. Diffuse neural cell death may be mediated by oxidative stress and glutamate-induced excitotoxicity after mTBI. Many studies have demonstrated that EX-4 has a neuroprotective function to minimize mTBI impairment, prevent cell death, and improve cognitive function [124,125,126]. Two studies by Greig’s NIH group reported that treatment with EX-4 prevents mTBI-induced gene expression associated with dementia and AD [126,127]. Liraglutide, another long-acting GLP-1 analogue, has been widely studied in TBI and determined to have neuroprotective effects similar to EX-4 [128,129]. 

Evidence from previous studies indicates that liraglutide functions to ameliorate memory impairment, augment anti-inflammatory processes, reduce cerebral edema in the pericontusional regions, improve sensorimotor function, preserve blood–brain barrier integrity, and reduce cortical tissue loss in neuronal cultures and the mTBI mouse model [128,129]. In 2015, Greig’s NIH group reported that pretreatment with liraglutide rescued neuronal cells from oxidative stress- and glutamate excitotoxicity-induced cell death that mimicked the injured condition in the post-mTBI brain. The same study suggested that the cAMP/PKA/pCREB signaling pathway has an important role in this neuroprotective activity of liraglutide [129]. The same group recently demonstrated that treatment with twincretin, another agonist of GLP-1R, enhanced viability of glutamate- and hydrogen peroxide-treated SH-SY5Y cells and protected against 6-hydroxydopamine-induced injury in rat primary cultures of dopaminergic ventral mesencephalon neurons [130]. This study also revealed that administration of twincretin restores the visual and spatial memory deficits induced by mTBI in the mouse model. The neuroprotective effects of twincretin were suggested to be provided via the CREB-mediated signaling pathway [130].

Sitagliptin, a DPP-4 inhibitor, is a clinic drug for type 2 diabetes mellitus and could potentially be a treatment for TBI also. The DPP-4 inhibitors have not been extensively investigated for TBI treatment. A recent study from DellaValle et al. [131] demonstrated that oral administration of sitagliptin reduces brain lesion size and brain cell death via activation of the CREB-mediated signaling pathway in the TBI mouse model; moreover, CREB-regulated expression of manganese superoxide dismutase (MnSOD) was increased in sitagliptin-treated mice. All these lines of evidence from previous studies indicate agonists of GLP-1R have potential as pharmacological-based therapies for TBI.

### 3.3. Stimulation of GLP-1R and Alzheimer’s Disease

Starting in the late 1990s, GLP-1R gained attention as a potential therapeutic target for AD, since diabetes and AD share similar pathological features, including chronic oxidative stress and inflammatory responses. GLP-1 prevented murine hippocampal HT22 cells from cell death by treatment with H_2_O_2_-, Aβ_1–42_, and other toxic agents through AKT- and ERK1/2-mediated signaling pathways [132]. The long-lasting GLP-1 analogue, EX-4, markedly rescued learning and memory deficits, stimulated long-term potentiation (LTP) via the cAMP-CREB signaling axis, and regulated intracellular Ca^2+^ homeostasis in the Aβ fragment-induced rat hippocampal injury model [133,134]. Liraglutide, a long-acting GLP-1R agonist, was extensively explored as a therapeutic agent for AD. Previous studies on AD animal models demonstrated that liraglutide significantly reduces neuronal hyperphosphorylated tau, prevents decline in learning and memory, increases protein O-glycosylation, halts loss of hippocampal neurons, decreases Aβ plaque load, and prevents synaptic loss [135,136,137,138]. In addition, an in vitro study revealed that the neuroprotective function of liraglutide is mediated through the PI3K-AKT signaling pathway [139]. Lixisenatide, another GLP-1R agonist, developed to treat type 2 diabetes, has been shown to have neuroprotective effects similar to liraglutide, including improved working memory, increased LTP, decreased Aβ deposition, and reduced inflammatory responses in an AD mouse model [140]. These neuroprotective effects of lixisenatide were attributed to induced AKT and MEK signaling pathways [141].

The DDP-4 inhibitor linagliptin has been reported to possess the neuroprotective functions of attenuating Aβ plaque formation, preventing GSK3β and tau hyperphosphorylation, alleviating inflammation, and increasing brain incretin levels. Furthermore, linagliptin mitigates Aβ-induced mitochondrial dysfunction and intracellular ROS production by stimulating 5′ AMP-activated protein kinase (AMPK)-Sirt1 signaling [142,143].

### 3.4. Stimulation of GLP-1R and Parkinson’s Disease

Current treatments of PD are targeted on defective motor function, which is a consequence of dopamine deficiency. In the MPTP-induced PD mouse model, EX-4 treatment protected dopaminergic neurons against degeneration, preserved dopamine levels, and improved motor function [144]. Two studies of Harkavyi et al. reported that administration of EX-4 increased cellular BrdU incorporation in the rat subventricular zone (SVZ) and substantia nigra (SN), and markedly elevated striatal dopamine concentration in the 6-hydroxydopamine (6-OHDA)-induced PD animal model [145,146]. These results suggest that EX-4 can protect neurons against metabolic and oxidative insults, and provide preclinical support for the therapeutic potential of EX-4 in the treatment of PD. Moreover, vildagliptin, a DPP-4 inhibitor, blocked the receptor for advanced glycation end products (RAGE) activated-NF-κB pro-inflammatory signaling cascade, prevented dopaminergic neuron death, and ameliorated motor impairment in the rat rotenone model of PD [147].

The first open-label clinical study reported that administration of exenatide, a long-acting GLP-1R agonist, showed long-lasting improvements in motor and cognitive function in PD patients [148]. However, liraglutide, another GLP-1R agonist, revealed no neuroprotective effects in the context of moderate or substantial midbrain dopaminergic neuronal loss and associated functional motor deficits in the rat 6-OHDA lesion model of PD [149]. The underlying cause for the discrepancy in the protective effect in PD models needs to be further clarified.

### 3.5. Stimulation of GLP-1R and Huntington’s Disease

There are extensive studies of GLP-1 and neurodegenerative diseases but studies related to HD have been limited until recently, perhaps because HD is relatively rare compared to other neurodegenerative diseases for epidemiological comparison. HD shares similar pathogenesis and etiological complexity with other neurodegenerative disorders. Agonists of GLP-1R and DPP-4 inhibitors may exert their neuroprotective functions in HD as well. Using the website ClinicalTrials.gov (Available online: https://clinicaltrials.gov/) (A service of the U.S. National Institutes of Health), we searched for studies with “GLP-1 and Huntington’s disease” and no results were found. When we changed the search term to “Huntington’s disease”, 143 studies were found but none of these cases related to GLP-1 or its analogues. In addition, we searched “GLP-1 and neurological disorders” and found 47 cases, some of them studying AD and PD, but HD was still not found in the search.

There are limited studies concerning GLP-1/GLP-1R and HD. Nevertheless, a few studies have reported that administration of GLP-1-Tf (GLP-1 fused to human transferrin) or EX-4 improved pancreatic morphology, peripheral glucose regulation, motor coordination, and extended life span as a consequence of significantly altered hypothalamic gene transcription signatures and energy metabolism in HD mouse models [34,150]. EX-4 treatment also suppressed cellular pathology, aggregation of mutant htt protein, in both the brain and pancreatic islet in HD mice [34]. The studies implied that GLP-1 and its analogues are tentative agents for therapeutic intervention for HD patients

## 4. Emerging Role of GLP-1 Receptors in DNA Repair

GLP-1R initiated neuroprotective effects have been reported to involve many cellular processes, as described in Section 3. However, very few studies have reported the correlation between GLP-1 and DNA repair in neurological disorders. Therefore, we discussed the role of APE1, the pivotal protein in the major repair pathway for oxidative DNA damage involved in neurological diseases. We also reviewed the neuroprotective effects and mechanisms of activated GLP-1R in the previous two sections.

**Figure 2:**
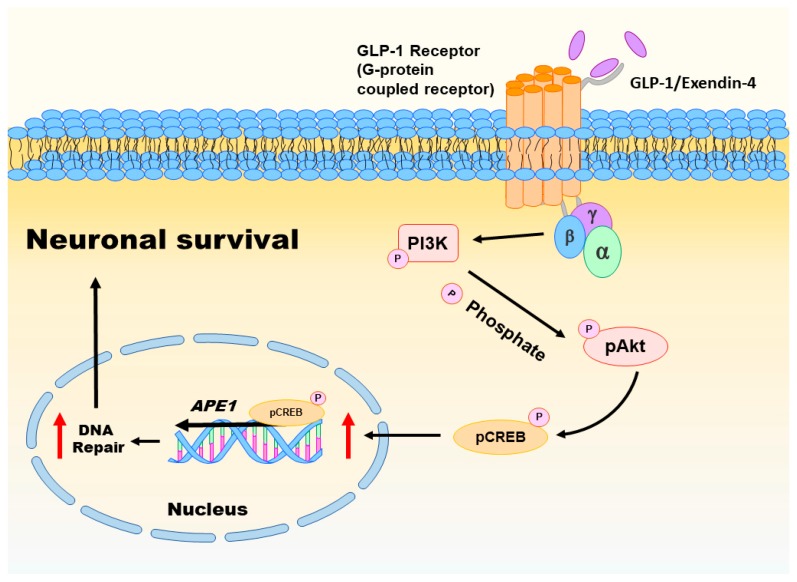
This schematic diagram shows the active GLP-1R induced mechanisms that enhance DNA repair efficiency and neuronal survival. The stimulation of GLP-1R enhances APE1 expression and increases DNA repair via the PI3K-AKT-mediated signaling pathway. The greater DNA repair efficiency provides better genomic integrity and greater neuronal viability. Red arrow: increased activity.

Oxidative injury of brain cells is a common form of pathogenesis in neurodegenerative diseases and brain traumas [151,152]. In a recent study [26], we employed menadione as a ROS inducer to generate oxidative insults to the vehicle or GLP-1/EX-4 treated rat primary cortical neurons. We found that GLP-1/EX-4 treated cortical neurons are more resistant to oxidative stress. Previous studies have demonstrated that menadione is capable of inducing oxidative DNA damage, as well as triggering apoptosis in neurons [27,153]. Thus, we postulated that activation of GLP-1R rescues neurons from menadione-induced oxidative insults through enhancing DNA repair efficiency and maintaining genomic integrity. Our study examined whether the proteins involved in the BER pathway were affected by GLP-1R-mediated signaling, including 8-oxoguanine DNA glycosylase (OGG1), nei endonuclease VIII-like 1 (NEIL1), flap endonuclease 1 (FEN1), uracil DNA glycosylase (UDG), APE1, DNA polymerase β (Polβ), and ligase III. None of the BER proteins and their enzymatic activities were significantly affected by the activation of GLP-1R, except APE1. The protein level of APE1 was upregulated 1.5-fold to 2-fold in GLP-1/EX-4 treated cortical neurons, compared with vehicle controls. In addition, the incision activity of APE1 was also elevated approximately 1.5-fold by GLP-1/EX-4 treatment. Furthermore, we used the middle cerebral artery occlusion (MCAO) reperfusion rat model to induce a cerebral ischemic stroke, in order to test whether stimulation of GLP-1R enhances DNA repair efficiency in brain cells. APE1 expression was higher in EX-4 treated MCAO animals than the vehicle treated MCAO group. Results of the TUNEL assay and γH2AX labeling both directly and indirectly demonstrated that administration of EX-4 effectively reduced nuclear DNA damage in MCAO animals. The results of our study suggested that better DNA repair capability is the consequence of upregulated APE1 via stimulation of GLP-1R (see Figure 2).

Activation of GLP-1R triggers two major downstream signaling pathways: MEK-ERK1/2 and PI3K-AKT [111]. Both signaling pathways activate CREB. Our previous study examined both signaling pathways in GLP-1/EX-4 treated cortical neurons. The results indicate that treatments of both GLP-1 and EX-4 promptly and significantly elevated phosphorylated AKT and CREB levels but phosphorylation of ERK1/2 was only slightly increased. Inhibitors of MEK and PI3K were utilized to determine the signals that regulate APE1 expression; the results indicated that the PI3K-AKT-CREB signaling axis is the main signaling pathway in the control of APE1 expression in reaction to GLP-1R activation in primary cortical neurons (see Figure 2) [26]. Evidence from our previous in vitro and in vivo studies suggested that stimulation of GLP-1R enhanced neuronal viability and DNA repair capability via PI3K-AKT-mediated APE1 expression. GLP-1R, therefore, is a potential candidate for developing innovative treatments in cerebral ischemic stroke and neurodegenerative diseases [26].

## 5. Conclusions

The pharmacological spectrum of GLP-1 and its analogues has been widely inspected for applications to many diseases, not just diabetes. As described in the previous sections, past studies have revealed that stimulation of GLP-1R ameliorates pathological symptoms of neuronal disorders, effectively improves cognitive and motor functions, and significantly enhances neuronal survival. Most of these studies suggest that neuroprotective functions of activated GLP-1R include suppressing inflammatory response, anti-oxidant processes, counteracting protein misfolding, enhancing neuronal genesis, and anti-apoptotic processes. Until now, relatively few studies have focused on DNA damage and repair as a treatment target for neurological disorders. We would like to promote the enhancement of DNA repair capability as one possible preventive and therapeutic approach in the study of neurodegenerative diseases.
